# Activation of D2 dopamine receptor-expressing neurons in the nucleus accumbens increases motivation

**DOI:** 10.1038/ncomms11829

**Published:** 2016-06-23

**Authors:** Carina Soares-Cunha, Barbara Coimbra, Ana David-Pereira, Sonia Borges, Luisa Pinto, Patricio Costa, Nuno Sousa, Ana J. Rodrigues

**Affiliations:** 1 Life and Health Sciences Research Institute (ICVS), School of Health Sciences, University of Minho, Braga 4710-057, Portugal; 2 ICVS/3B's–PT Government Associate Laboratory, Braga/Guimarães 4710-057, Portugal

## Abstract

Striatal dopamine receptor D1-expressing neurons have been classically associated with positive reinforcement and reward, whereas D2 neurons are associated with negative reinforcement and aversion. Here we demonstrate that the pattern of activation of D1 and D2 neurons in the nucleus accumbens (NAc) predicts motivational drive, and that optogenetic activation of either neuronal population enhances motivation in mice. Using a different approach in rats, we further show that activating NAc D2 neurons increases cue-induced motivational drive in control animals and in a model that presents anhedonia and motivational deficits; conversely, optogenetic inhibition of D2 neurons decreases motivation. Our results suggest that the classic view of D1–D2 functional antagonism does not hold true for all dimensions of reward-related behaviours, and that D2 neurons may play a more prominent pro-motivation role than originally anticipated.

## Abstract (web summary)

Striatal D1 and D2-receptor expressing neurons have been shown to have opposing effects on reward-related behaviours. Here the authors reveal that specific activation of both D1 and D2 neurons in the nucleus accumbens leads to increase in motivational drive in rodents.

## Graphical abstract

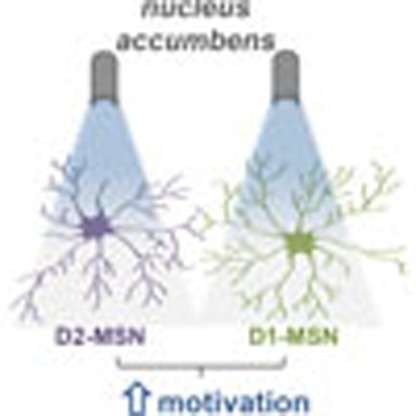

Dopaminergic neurotransmission in the nucleus accumbens (NAc) has an essential role in reward behaviours, although the causal biological contribution of this mechanism remains controversial. While some argue that dopamine signals the discrepancy between predicted and experienced reward (reward prediction error)^1^,^2^,^3^, others suggest that it mediates motivational drive by the attribution of incentive salience to reward-related stimuli^4^.

Up to 95% of NAc neurons are medium spiny neurons (MSN), typically segregated into those expressing D1 dopamine receptors (direct pathway) and those expressing D2 dopamine receptors (indirect pathway)^5^,^6^,^7^. The existence of a functional opposition between D1 and D2 MSN has for long been assumed^8^,^9^, but the establishment of a causal relationship between the activation of each neuronal subtype and its effect on complex motivated behaviours has proved to be challenging. D1-MSN activation is canonically related to positive rewarding events, inducing persistent reinforcement, whereas D2-MSN signalling is thought to mediate aversion (both in dorsal striatum and NAc)^10^,^11^,^12^. Nonetheless, recent studies raised some questions regarding this functional/behavioural bias, especially regarding D2 neurons in the NAc^13^,^14^,^15^. Taking this into consideration, we used an optogenetic approach to address the impact of modulating NAc D1 and D2 neurons in motivation-dependent behaviours.

## Results

### NAc D1 and D2 activation is correlated with motivation

**Figure 1:**
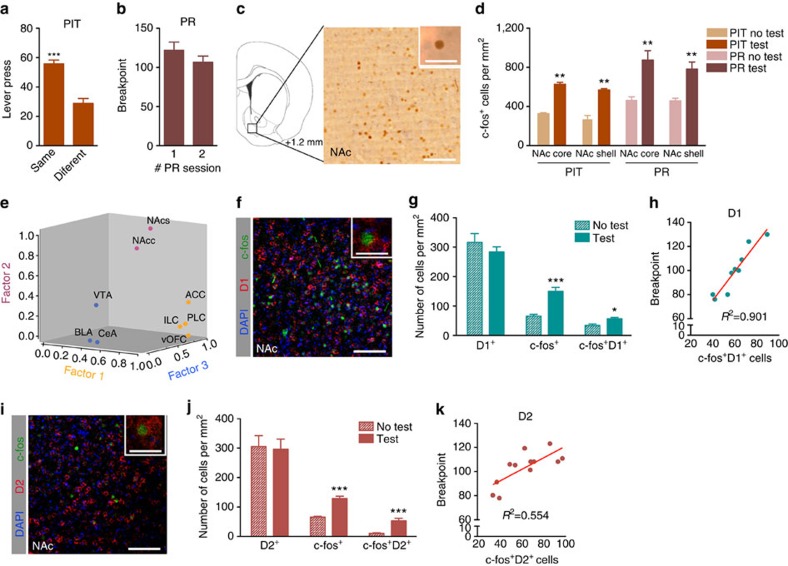
NAc D1 and D2 neuronal activation predicts performance in motivation-related tasks. (**a**) PIT outcome (*n*=10). (**b**) Breakpoint for two PR sessions (*n*=10). (**c**) Representative immunostaining of c-fos in the NAc; Scale bar: 100 μm; inset: 20 μm. (**d**) Animals performing PIT or PR tests have increased c-fos^+^ cells in the NAc (*n*=6). (**e**) Principal factor analysis was done to evaluate the contribution of each brain region for the behavioural performance. This analysis shows that the NAc core and shell regions (NAcc and NAcs; factor 2) are grouped distinctively from other limbic regions (BLA, CeA and VTA; factor 3) and from cortical regions that are all grouped together in factor 1 (ACC, PLC, ILC, lOFC and vOFC). Representative images of immunofluorescence for c-fos and dopamine receptor D1 (**f**) or c-fos and dopamine receptor D2 (**i**) in the NAc of animals that performed PR task; Scale bar: 100 μm; inset: 20 μm. Insets represent double positive cells. (**g**) Number of c-fos^+^/D1^+^ cells and c-fos^+^/D2^+^ (**j**) cells in the NAc (*n*=6). (**h**) Correlation between individual Breakpoint and number of c-fos^+^/D1^+^ cells in the NAc (*n*=9). (**k**) Correlation between individual breakpoint and number of c-fos^+^/D2^+^ cells in the NAc (*n*=12). Error bars denote s.e.m. *P≤0.05, **P ≤0.01, ***P≤0.001.

First, to verify that the NAc was critically recruited in motivation-dependent tasks, we evaluated the neuronal activation pattern of different brain regions after the Pavlovian-to-Instrumental transfer (PIT) and the Progressive-Ratio tasks (PR). PIT evaluates the ability of Pavlovian conditioned cues that predict reward to enhance instrumental response^16^,^17^, i.e., a measure of incentive salience, whereas the PR measures the willingness to work to obtain a reward (breakpoint)^18^. All corticolimbic regions analysed presented a significant increase in c-fos on task execution (Fig. 1a–d and Supplementary Fig. 1a–r; two-way analysis of variance (ANOVA), *P*<0.001, *n*=6). To further understand the contribution of each brain region for the behavioural performance, we performed an exploratory factor analysis through Principal Axis Factoring, which makes no *a priori* assumptions about relationships among factors. By defining three factors, this analysis clearly grouped all cortical regions in factor 1 (ACC: anterior cingulate cortex; PLC: prelimbic cortex; ILC: infralimbic cortex; lOFC and vOFC: lateral and ventral orbitofrontal cortex); factor 2 grouped NAc core and shell; and factor 3 included other limbic structures (CeA: central nucleus of the amygdala; BLA: basolateral amygdala; and ventral tegmental area (VTA)) (Fig. 1e). All communalities and factor loadings were satisfactory, revealing no problematical cross loadings. Cronbach's Alpha denotes good, or excellent, reliability scores (Supplementary Table 1). Pearson's correlation coefficients revealed that there is a positive association between the three factors and behavioural test score, although, only NAc (Pearson's correlation, *P*=0.002) and other limbic regions (Pearson's correlation, *P*=0.017) were significant. Using a multiple linear regression model, we verified that 46.9% of behavioural performance could be explained by the three predictors; however, only NAc was significant (*β*=0.5, *P*=0.042).

Considering previous results, we then measured the recruitment of NAc D1^+^ and D2^+^ neurons during PR performance. Importantly, there was a significant increase in both NAc c-fos^+^/D1^+^ and c-fos^+^/D2^+^ cells on PR performance (*n*=6; Fig. 1f–g, c-fos^+^/D1^+^: unpaired *t*-test, *P*<0.001; Fig. 1i,j, c-fos^+^/D2^+^: unpaired *t*-test, *P*=0.001). Both were positively correlated with individual breakpoint values (Fig. 1h, c-fos^+^/D1^+^: Pearson's correlation, *R*^2^=0.9, *P*<0.001, *n*=9; Fig. 1k, c-fos^+^/D2^+^: Pearson's correlation, *R*^2^=0.7, *P*=0.006, *n*=12).

### Absence of D1–D2 antagonism in motivation

**Figure 2:**
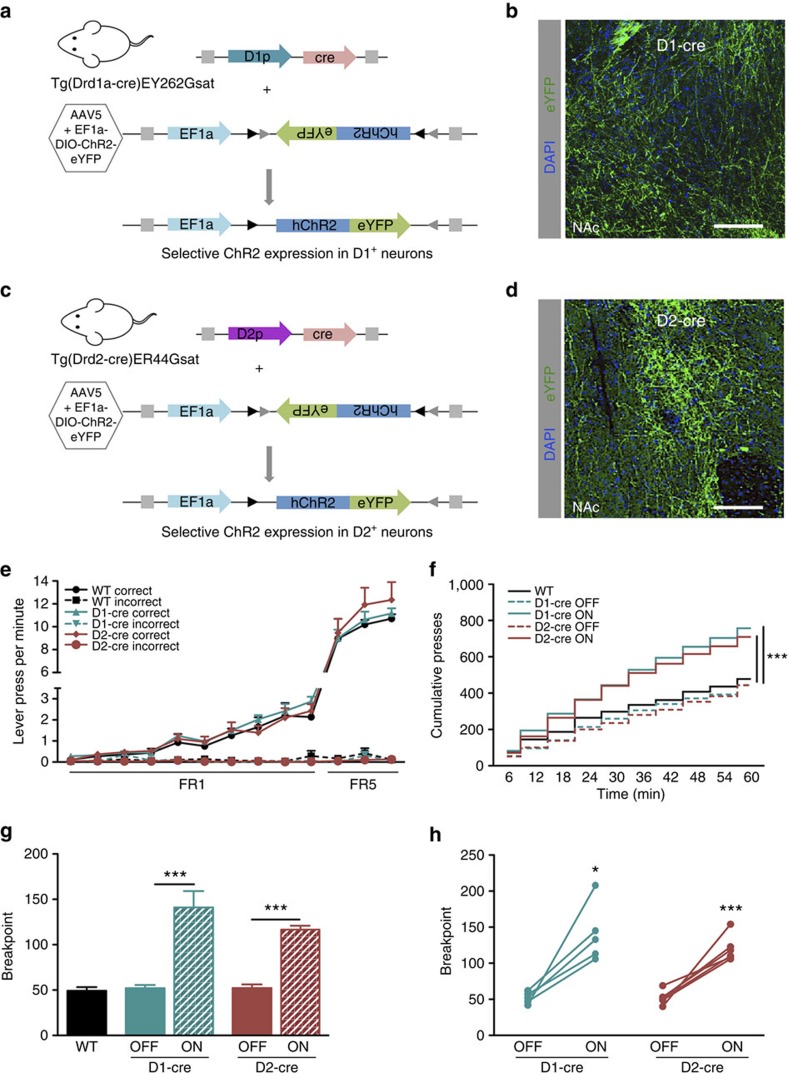
Optogenetic activation of NAc D1 and D2 neurons enhances motivation in mice. (**a**) Strategy used for optogenetic activation of NAc D1 neurons in mice. A cre-dependent ChR2 construct (AAV5-EF1a-DIO-ChR2-eYFP) was injected unilaterally in the NAc of D1-cre transgenic mice (Tg(Drd1a-cre)EY262Gsat). (**b**) Expression of ChR2-eYFP in D1-cre animals; Scale bar: 50 μm. (**c**) Strategy used for optogenetic activation of NAc D2 neurons in mice. A cre-dependent ChR2 construct (AAV5-EF1a-DIO-ChR2-eYFP) was injected unilaterally in the NAc of D2-cre transgenic mice (Tg(Drd2-cre)ER44Gsat). (**d**) Expression of ChR2-eYFP in D2-cre animals; scale bar: 50 μm. (**e**) No differences were found in the learning curves of PR test between groups (n_D1-cre_=5; n_D2-cre_=6). (**f**) Optogenetic activation of D1 or D2 neurons (12.5 ms light pulses at 40 Hz, during 1 s of cue exposure) increased cumulative presses throughout session. (**g,h**) Optogenetic activation of NAc D1 or D2 neurons strongly enhanced breakpoint. Error bars denote s.e.m. *P≤0.05, ***P≤0.001.

Since both D1 and D2 neurons were activated during PR, we decided to causally test the effect of cell type-specific activation on motivated behaviour. For this purpose, we injected a cre-inducible adeno-associated viral construct coding for channelrhodopsin (ChR2) (pAAV-EF1a-DIO-hChR2-enhanced green fluorescent protein (eYFP)) unilaterally in the NAc of D1 and D2-cre transgenic mouse lines and subjected animals to PR task (Fig. 2a–d). No differences in training were observed between groups (Fig. 2e). As anticipated, activation of D1 neurons during cue exposure increased cumulative presses throughout session as well as the breakpoint (Fig. 2f–h; 85% increase; paired *t*-test, *P*=0.011, *n*=5). Surprisingly, activation of D2 accumbal neurons also enhanced cumulative presses and breakpoint (Fig. 2f–h; 69% increase; paired *t*-test, *P*=0.003, *n*=6; results from other stimulation parameters in Supplementary Fig. 2).

### Exploring the role of D2 neurons in motivation

**Figure 3:**
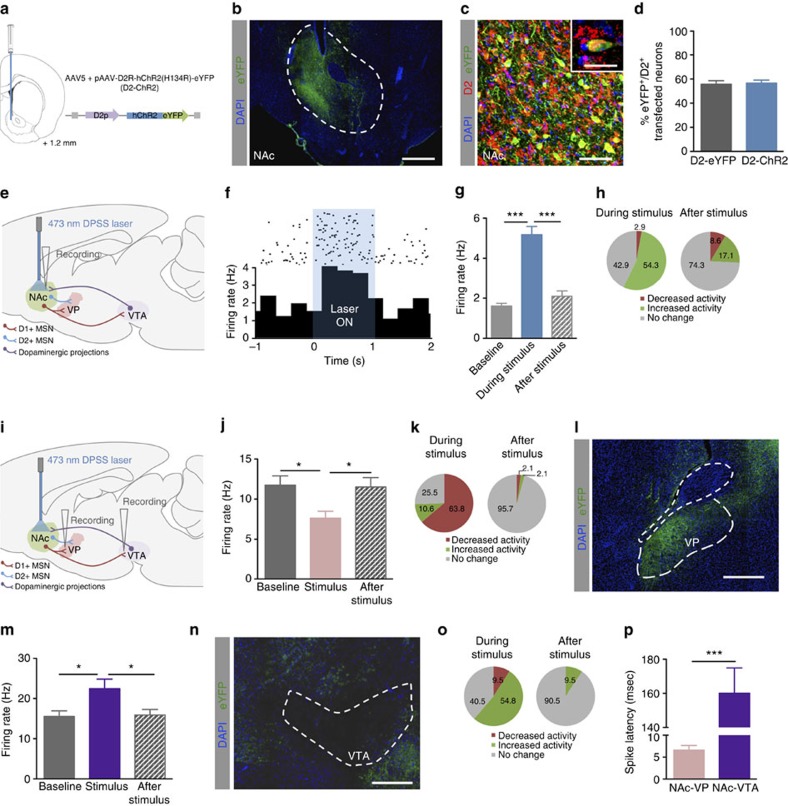
*In loco* and downstream electrophysiological recordings. (**a**) Rats received unilateral injection of AAV5-D2-hChR2(H134R)-eYFP in the NAc; number represent distance to bregma. (**b**) Immunofluorescence against YFP showing transfection restricted to the NAc; scale bar: 200 μm; ac: anterior commissure. (**c**) Immunofluorescence showing expression of eYFP in D2^+^ neurons; scale bar: 200 μm; inset: 40 μm. (**d**) Around 58% of D2^+^ neurons express D2-eYFP or D2-ChR2 (*n*=6). (**e**) Schematic representation of the optogenetic stimulation and *in vivo* single-cell electrophysiological recording experiments. (**f**) Representative time histogram of NAc electrophysiological single units in response to a 40 Hz stimulus. (**g**) Increase in NAc firing rate during optogenetic stimulation (40 Hz, 12.5 ms pulses for 1 s). (**h**) Approximately half of the cells increased firing rate (54%), 42.9% did not respond and 2.5% decreased their activity (*n*=19 cells). (**i**) Schematic representation of the *in vivo* single-cell electrophysiological recording experiments in downstream regions. (**j**) NAc D2 neuronal activation decreases global VP firing rate (*n*=30 cells). (**k**) During stimulation, 63.8% of VP cells decrease their firing rate, consistent with GABAergic inputs from D2 MSNs from the NAc, 25.5% do not respond and 10.6% increased activity. Considering a 60 speriod after the stimulation, most of the cells return to their basal activity levels. (**l**) eYFP expression in the VP showing that D2-ChR2^+^ terminals arising from the NAc strongly innervate this brain region; scale bar: 500 μm. (**m**) NAc D2 neuronal stimulation increases global VTA firing rate (*n*=25 cells). (**n**) During stimulation, 54.8% of cells increase their firing rate, 40.5% do not respond and 9.5% decreased activity; most of the cells return to their basal activity levels after stimulation. (**o**) eYFP expression in the VTA showing rare D2-ChR2^+^ terminals; scale bar, 500 μm. (**p**) Spike latency after NAc D2 optogenetic stimulation shows that VP cells fire almost immediately, consistent with a monosynaptic input from the NAc whereas VTA cells present higher latency (indirect/polysynaptic modulation). Error bars denote s.e.m. *P≤0.05, ***P≤0.001.

While the behavioural results obtained with D1 neuronal activation were expected, D2 activation effects in motivation were paradoxical, in the light of previous findings suggesting that D2 neurons are associated with negative rather than positive valence events^10^,^12^,^19^. Considering this, we decided to further explore the role of NAc D2 neurons in behaviour. We injected in the NAc of rats a construct carrying ChR2 under the control of the D2 receptor minimal promoter—pAAV-D2R-hChR2(H134R)-eYFP (Fig. 3a,b)—to allow specific optogenetic activation of D2^+^ neurons. 58% of NAc D2^+^ neurons were successfully transfected with D2-driven ChR2 or EYFP (D2^+^/YFP^+^ cells; Fig. 3c,d); we rarely observed D1^+^/YFP^+^ cells (1.5%; Supplementary Fig. 3a–b). By evaluating the optically evoked response using single-cell *in vivo* electrophysiology, we showed that D2-driven opsin was functional at different stimulation frequencies (Fig. 3e–g; two-way ANOVA, *P*<0.001, *n*=19 cells; Supplementary Fig. 3c). Approximately half of the NAc cells (54.3%) increased their firing rate during 40 Hz stimulation (42.9% did not respond and 2.9% decreased activity); and 25.7% of the cells presented altered firing rate during the 60 s after the stimulus (8.6% decreased activity, 17.1% increased activity; Fig. 3h).

To confirm specificity of D2 pathway, we performed electrophysiological recordings in the ventral pallidum (VP) (Fig. 3i), which receives direct projections from both D1 and D2 NAc MSNs^13^,^20^. NAc D2 neuronal stimulation elicited an overall reduction in the firing rate of the VP (Fig. 3j,k; one-way ANOVA, *P*<0.001, *n*=30 cells), with an average spike latency of 6 ms (Fig. 3p), consistent with the expected monosynaptic (GABAergic) input from the NAc to VP. Because it has been shown that only D1^+^ (but not D2^+^) MSNs project directly to the midbrain^13^,^21^,^22^, we also recorded the VTA during D2 neuronal activation in the NAc. Confirming the selectivity of D2 pathway, we observed a general increase in global VTA activity (Fig. 3m,n; one-way ANOVA, *P*<0.001, *n*=25 cells), with an average spike latency of 160 ms, suggestive of indirect modulation (Fig. 3p). These results were further confirmed by recording experiments of these downstream regions during stimulation of NAc terminals (Supplementary Fig. 4). Moreover, immunofluorescence against YFP showed that VP (but not VTA) is directly innervated by D2-ChR2 terminals (Fig. 3l,o).

**Figure 4:**
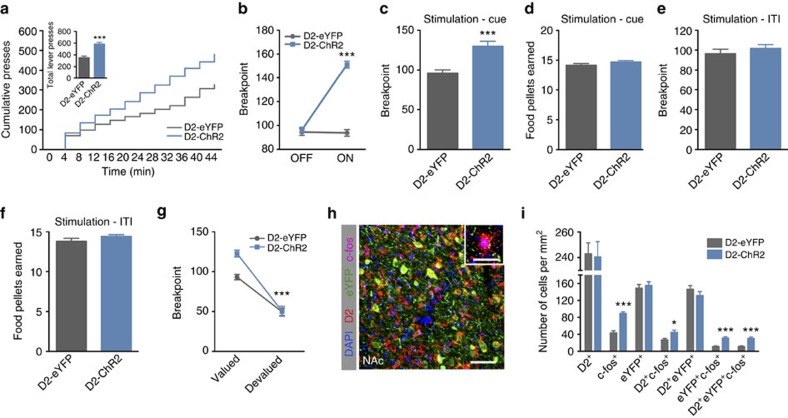
Optogenetic activation of NAc D2 neurons increases motivation in rats. (**a**) Optogenetic stimulation of D2-ChR2 animals during cue exposure (40 Hz, 12.5 ms pulses for 1 s) increases cumulative presses in the PR test (n_D2-ChR2_=16; n_D2-eYFP_=13). (**b**,**c**) D2 activation during cue exposure induces a significantly higher breakpoint; though animals receive the same number of pellets in the session (**d**). (**e**) D2 activation during inter-trial interval (ITI) does not change breakpoint (n_D2-ChR2-eYFP_=7; n_D2-eYFP_=5). (**f**) Total number of food pellets earned in the PR session, in which optical stimulation was given during the ITI. (**g**) Outcome devaluation decreases lever presses in both groups. (**h**) Representative immunofluorescence for c-fos^+^/D2^+^/eYFP^+^ in the NAc; scale bar: 200 μm; inset: 40 μm. (**i**) Quantification of c-fos^+^/D2^+^/eYFP^+^ cells in the NAc of stimulated animals showing the recruitment of D2^+^/eYFP^+^ cells (*n*=5 animals). Error bars denote s.e.m. *P≤0.05, ***P≤0.001.

We next tested the effects of NAc D2 neuronal activation in the PR task. All groups acquired similar levels of lever pressing during training (Supplementary Fig. 3d–e). D2 optical stimulation (473 nm light; pulses of 12.5 ms at 40 Hz for 1 s) occurring at the same time as the unconditioned stimulus (light above the active lever), induced a significant increase in cumulative presses throughout the session, which translated into a 35% enhancement of the breakpoint (Fig. 4a–c; unpaired *t*-test, *P*<0.001, *n*=13–16; Supplementary Fig. 3f,g shows PR with different stimulation protocols). This enhancement was not due to changes in the number of pellets earned in the session (Fig. 4d). Importantly, when this stimulation occurred during the inter-trial interval (ITI), it had no effect (Fig. 4e,f; unpaired *t*-test, *P*=0.395, *n*=5–7). In an additional PR session (with optical stimulation), animals were satiated to induce reward devaluation. Both groups decreased lever pressing, demonstrating sensitivity to the decreased value of the outcome, albeit this was more evident in D2-ChR2 group (Fig. 4g, two-way ANOVA, *P*<0.001). Optical stimulation of NAc D2 neurons during the conditioned stimulus presentation also robustly enhanced PIT performance, despite similar baseline performance (Supplementary Fig. 5a–d; paired *t*-test, *P*=0.01). No changes were observed in feeding behaviour or locomotion of stimulated animals (Supplementary Fig. 5e–f).

Confirming the behavioural findings, the number of c-fos^+^/D2^+^/eYFP^+^ cells in the NAc was higher in stimulated D2-ChR2 rats when compared with stimulated D2-eYFP control rats on PR task (Fig. 4h,i; unpaired *t*-test, *P*=0.001, *n*=5 animals).

**Figure 5:**
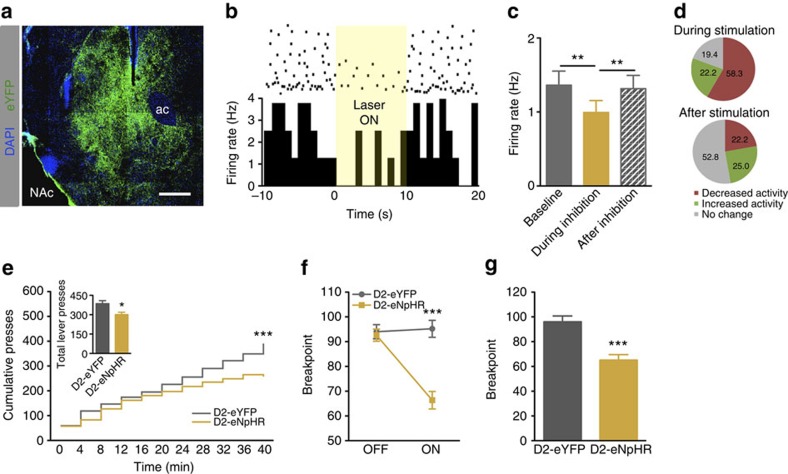
Inhibition of NAc D2 neurons - electrophysiological and behavioural correlates. (**a**) Representative immunofluorescence against YFP in the NAc of an animal injected with AAV5-D2-eNpHR-eYFP. Scale bar: 400 μm; ac: anterior commissure. (**b**-**c**) Optogenetic stimulation of D2-eNpHR (10 s constant light at 15 mW) decreases NAc firing rate (*n*=20 cells). (**d**) During stimulation, 58.3% of cells decrease their firing rate, whereas 22.2% of cells increase their activity and 19.4% do not respond. After the stimulation (period of 60 s), 52.8% of cells return to their basal activity levels, 22.2% of cells present decreased firing rate, and 25% of cells present increased activity. (**e**) Optogenetic inhibition of D2 neurons during cue exposure (15 mW constant light during 10 s) decreases cumulative presses and breakpoint (**f**,**g**) in the PR test (n_D2-ChR2_=8; n_D2-eYFP_=5). Error bars denote s.e.m. **P ≤0.01, ***P≤0.001.

Our next goal was to understand the impact of inhibiting D2^+^ accumbal neurons in motivation. To do so, a vector containing halorhodopsin (NpHR) under the control of D2 minimal promoter (pAAV-D2R-NpHR-eEYFP) was injected unilaterally in the NAc (Fig. 5a). Optical activation of NpHR (589 nm laser; 10 s constant light at 5mW) lead to a general decrease in the NAc neuronal firing rate (Fig. 5b,c; one-way ANOVA, *P*=0.0015, *n*=20 cells), with 58.3% of the cells presenting decreased activity and 22.2% increased activity, while 19.4% did not respond (Fig. 5d). At a behavioural level, optical inhibition of NAc D2 neurons during cue exposure decreased cumulative lever presses (Fig. 5e; two-way ANOVA, *P*<0.000) and strongly reduced breakpoint (Fig. 5f,g; 32% decrease, unpaired *t*-test, *P*=0.001, *n*=5-8). No significant effects in locomotion and feeding behaviour were observed due to D2 optogenetic inhibition (Supplementary Fig. 6).

### Rescue of deficits in a prenatal glucocorticoid exposure model

**Figure 6:**
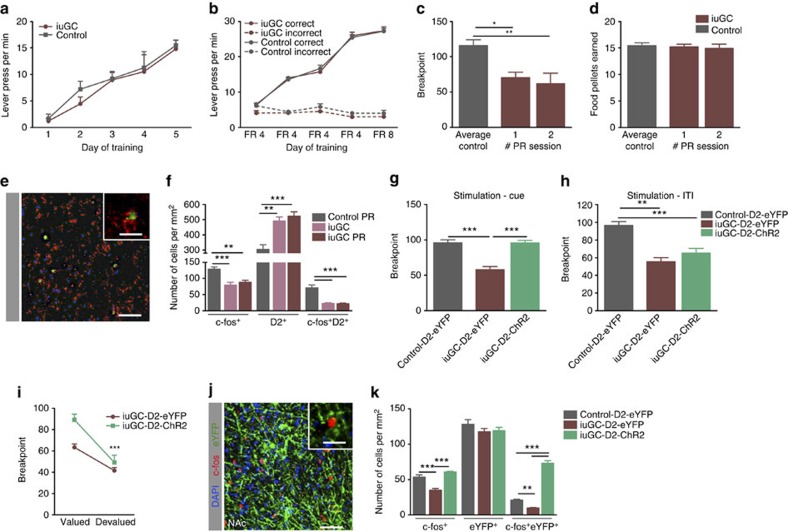
NAc D2 neuronal activation rescues the behavioural deficits in a model of D2 dysfunction. (**a**) Continuous reinforcement (CRF) training sessions of the PR test shown as average number of lever presses per minute (*n*=10). (**b**) Fixed ratio (FR) training sessions of the PR test are shown as average number of lever presses per minute. (**c**) iuGC rats have a lower breakpoint when compared with control animals; though the total number of food pellets earned in the PR test sessions is similar (**d**). (**e**) Representative image of double immunofluorescence for c-fos and D2 in the NAc; scale bar: 200 μm; inset: 40 μm. (**f**) iuGC animals present a reduction in the recruitment of D2 neurons (c-fos^+^/D2^+^ cells) (*n*=6); iuGC animals present reduced total c-fos^+^ cells and increased D2^+^ cells in the NAc, regardless of having performed the PR task or not. (**g**) D2 optogenetic stimulation during cue exposure normalizes iuGC breakpoint (iuGC-D2-ChR2 rats) (n_control-D2-eYFP_=11; n_iuGC-D2-ChR2_=15; n_iuGC-D2-eYFP_=13). (**h**) When the optical stimulation was given during inter-trial interval (ITI), no effect in motivation was observed (n_control-D2-eYFP_=7; n_iuGC-D2-ChR2_=7; n_iuGC-D2-eYFP_=5). (**i**) Breakpoint before and after food devaluation is depicted as average of absolute value; both groups decrease lever presses in devalued condition. (**j**) Representative image of immunofluorescence for c-fos and eYFP in the NAc of an animal that performed the PR task. Scale bar: 200 μm; inset: 40 μm. (**k**) Quantification of c-fos^+^ and eYFP^+^ cells in the NAc after PR performance with optogenetic stimulation shows that ChR2 neurons are being activated (*n*=6); stimulated iuGC-D2-ChR2 animals present a substantial increase in the number of c-fos^+^/eYFP^+^ cells in comparison to stimulated D2-eYFP animals. Error bars denote s.e.m. *P≤0.05, **P≤0.01, ***P≤0.001.

We then assessed whether D2 accumbal stimulation was sufficient to normalize motivation in a rat model of *in utero* glucocorticoid exposure (iuGC) that presents hypodopaminergia in the mesolimbic circuit^23^,^24^, NAc D2 dysfunction and a significant impairment in motivation^15^. Control and iuGC animals were unilaterally injected with D2-driven ChR2 construct and subjected to the PR and PIT tasks. In the PR task, training was similar between groups (Fig. 6a,b; *n*=10). On the test day, iuGC rats presented a 53% decrease in their breakpoint (Fig. 6c; 62.5±6.4 versus 117±8.9; one-way ANOVA, *P*=0.003), despite consuming the same number of pellets in the session (Fig. 6d). Concordantly, these animals present an overall lower neuronal activation in the NAc when compared with control animals that also performed the test (Fig. 6e,f; one-way ANOVA, *P*=0.001, *n*=6); importantly, there was a marked reduction in the recruitment of D2^+^ neurons (unpaired *t*-test, *P*=0.001).

Optogenetic activation of D2 neurons during the PR test session significantly increased the breakpoint of iuGC animals (Fig. 6g; unpaired *t*-test, *P*<0.000, *n*=11–15), comparable to the one of control rats (iuGC-D2-ChR2 average of 95.85±4.5 versus control-D2-eYFP average of 96.1±4.1; unpaired *t*-test, *P*=0.97). In agreement, the number of c-fos^+^/eYFP^+^ positive neurons was also significantly higher than non-stimulated iuGC animals (Fig. 6j,k; unpaired *t*-test, *P*<0.001, *n*=6). Importantly, the behavioural rescuing effect was not observed if the stimulation occurred during the ITI (Fig. 6h). We also tested animals' response after reward devaluation in an additional PR session. In this scenario, both control and iuGC groups decreased lever pressing, demonstrating sensitivity to the decreased value of the outcome (Fig. 6i, one-way ANOVA, *P*<0.001, *n*=5–7).

In further support of an important role of D2 neurons in iuGC motivational deficits, we found that in the PIT test, optical stimulation of NAc D2 neurons also normalized the response of iuGC group (Supplementary Fig. 7).

## Discussion

Performance in motivation-related tasks has been tightly linked to the NAc: enhancing dopamine transmission in this region increases the willingness of animals to work for food^25^,^26^,^27^, whereas blunting dopamine signals attenuates it^26^,^28^,^29^. However, the function of D1 and D2 neurons is still debatable. Traditional views propose that tonic dopamine release mainly activates high-affinity D2 receptors, while phasic dopamine events activate low-affinity D1 receptors^30^,^31^, and signal the value of learned reward-predictive cues that drive-motivated behaviour^32^,^33^. However, recent findings show that phasic dopamine release also drives a rapid activation of D2 MSNs^34^, suggesting that some of the behavioural effects of phasic release may be partially mediated by D2 signalling.

Here we report that activation of both D1 and D2 neurons in the NAc is strongly correlated with behavioural performance in motivation-dependent paradigms. In addition, we show that brief optogenetic activation of NAc D1 and D2 neurons during reward-predictive cues strongly augments motivation in mice. Our findings are clearly distinct from the classic view supporting a functional opposition between D1 and D2 MSNs, indicating that both neuronal subtypes can contribute in the same direction in the modulation of motivation. Other optogenetic approaches specifically targeting NAc D1 and D2 neurons showed that D1 neuronal activation resulted in enhancement in conditioned place preference for cocaine, while the activation of D2 neurons attenuated preference, leading to the hypothesis that the D1 pathway has an action in promoting (drug) reward responses, while D2 pathway mediates the opposite^10^. These counterintuitive results may indicate that activation of D1 and D2 neurons in different behavioural contexts or during different stages of the task may lead to distinctive outcomes. In addition, these neurons may differentially modulate the response towards natural rewards versus drugs of abuse^35^.

Other D1/D2 optogenetic studies using natural rewards/reinforcers relied on the modulation of dorsomedial striatal neurons. Activation of D1 MSNs resulted in positive responses to rewarding stimuli, contrary to activation of D2 MSNs that induced aversion and suppression of reward/motivated behaviour in both place preference and operant tasks^11^. Our contradictory results regarding D2 neurons are probably the result of anatomical/functional specificities between the ventral and dorsal striatum. Indeed, a recent study showed that, in contrast to dorsal striatum, both NAc MSNs populations may inhibit or disinhibit thalamic activity depending on their projection pattern and not on their genetic (D1 or D2) characteristics^13^, emphasizing the need to revisit the current view of dorsal and ventral D1 and D2 MSNs as identical entities.

The unexpected behavioural outcome of D2 neuronal activation was further explored by selectively manipulating D2 neurons using a distinct methodological approach in rats. Akin to results in mice, brief activation (or inhibition) of NAc D2 neurons during reward-predictive cues strongly enhances (or diminishes) motivation. These findings were further extended by showing that activating accumbal D2 neurons in a model of prenatal glucocorticoid exposure that presents D2 dysfunction^15^,^23^ rescues motivation deficits of these animals.

It is important to stress that up to 80% of NAc cholinergic interneurons also express D2 dopamine receptor^36^,^37^,^38^,^39^, and their selective activation enhances phasic dopamine release in the NAc, which may ultimately synergize to drive-motivated behaviours^40^,^41^. However, cholinergic interneurons do not seem to be differently recruited between eYFP- and ChR2-stimulated animals (Supplementary Fig. 8), so the observed motivational effect seems to be mostly mediated by the activation of D2 MSNs of the NAc. This can arise through D2 MSNs direct modulation of VP activity, a brain region suggested to be a convergent point for motivational (and hedonic) signals^42^. Likewise, the observed indirect activation of VTA neurons (via VP or not) may also contribute to the observed motivation augmentation^43^,^44^,^45^. Interestingly, VP also reciprocally modulates NAc activity^46^. Thus, a complex cascade of neuromodulatory events can arise due to NAc D2 neuronal activation, highlighting the need to perform additional studies to better comprehend how activation of D2 (and D1) neurons can enhance motivated behaviour.

In conclusion, herein we show that different motivation-dependent tests recruit D1 and D2 neurons in the NAc, and that selective optogenetic activation of both neuronal populations enhances cue-induced motivational drive, suggesting that the classic view of D1–D2 functional antagonism does not hold true for all dimensions/types of reward-related behaviour.

## Methods

### Animals

*Rats*. Male Wistar Han rats (age of 2 months old at the beginning of the tests) were used. Animals were maintained under standard laboratory conditions: an artificial 12-h light/dark cycle (lights on from 08:00 to 20:00 hours), with an ambient temperature of 21±1 °C and a relative humidity of 50–60%; rats were individually housed after cannula implantation; standard diet (4RF21, Mucedola SRL) and water were given *ad libitum*, except when stated otherwise.

In the case of the model of iuGC, pregnant Wistar han rats (age of 9–11 weeks) were individually housed under the same standard laboratory conditions and food (4RF25, Mucedola SRL) and water were provided *ad libitum*. Subcutaneous injections of dexamethasone (Sigma) at 1 mg kg^−1^ (iuGC animals) or sesame oil (control group) were administered on gestation days 18 and 19. On postnatal day 21, male progeny was weaned and maintained under standard laboratory conditions. Male offspring derived from at least four different litters were used.

*Mice*. Male and female C57/Bl6 transgenic and non-transgenic mice (age of 2 months at the beginning of the tests) were housed at weaning in groups of five animals per cage. The progeny produced by mating D1-cre (Drd1a-cre, 262, Gensat) or D2-cre (Drd2-cre, ER44, Gensat) heterozygous transgenic male mice with wild-type C57/Bl6 females were genotyped at weaning by PCR. All animals were maintained under standard laboratory conditions: an artificial 12-h light/dark cycle (lights on from 08:00 to 20:00 hours), with an ambient temperature of 21±1 °C and a relative humidity of 50–60%; the mice were given a standard diet (4RF25 during the gestation and postnatal periods, and 4RF21 after weaning, Mucedola SRL) and water *ad libitum*.

All behavioural experiments were performed during the light period of the light/dark cycle. Health monitoring was performed according to FELASA guidelines^47^, confirming the Specified Pathogen Free health status of sentinel animals maintained in the same animal room. All procedures were conducted in accordance with European Regulations (European Union Directive 2010/63/EU). Animal facilities and the people directly involved in animal experiments were certified by the Portuguese regulatory entity—Direção Geral de Veterinária. All protocols were approved by the Ethics Committee of the Life and Health Sciences Research Institute.

*Mouse genotyping*. DNA was isolated from tail biopsy using the Citogene DNA isolation kit (Citomed). In a single PCR genotyping tube, the primers Drd1a F1 (5′-GCTATGGAGATGCTCCTGATGGAA-3′) and CreGS R1 (5′-CGGCAAACGGACAGAAGCATT-3′) were used to amplify the D1-cre transgene (340 bp), and the primers Drd2 (32108) F1 (5′-GTGCGTCAGCATTTGGAGCA-3′) and CreGS R1 (5′-CGGCAAACGGACAGAAGCAT-3′) to amplify the D2-cre transgene (700 bp). An internal control gene (lipocalin 2, 500 bp) was used in the PCR (LCN_1 (5′-GTCCTTCTCACTTTGACAGAAGTCAGG-3′) and LCN_2 (5′-CACATCTCATGCTGCTCAGATAGCCAC-3′). Heterozygous mice were discriminated from the wild-type mice by the presence of two amplified DNA products corresponding to the transgene and the internal control gene. Gels were visualized with GEL DOC EZ imager (Bio-Rad, Hercules, CA, USA) and analysed with the ImageLab 4.1 (Bio-Rad).

*Rat Behaviour and apparatus*. Rats were placed and maintained on food restriction (∼7 g per day of standard lab chow) to maintain 90% free-feeding weight. 45 mg food pellets (F0021; BioServ), used in the behavioural protocol, were placed in their home cages on the day before the first training session to familiarize the rats with the food pellets. Behavioural sessions were performed in operant chambers (Med Associates). Each chamber contained a central, recessed magazine that provided access to 45 mg food pellets (Bio-Serve) or 100 μl of sucrose solution (20% wt per vol in water) delivered by a pellet dispenser and a liquid dipper, respectively, two retractable levers with cue lights located above them that were located on each side of the magazine. Magazine entries were measured automatically by an infrared beam located at the entry of the magazine. A 1 kHz tone and an amplified white noise, each with a sound of 80 dB, where available as discrete auditory cues. A 2.8 W, 100 mA house light positioned at the top centre of the wall opposite to the magazine provided illumination. A computer equipped with Med-PC software (Med Associates) controlled the equipment and recorded the data.

*Rat PR schedule of reinforcement*. All training sessions started with illumination of the house light that remained until the end of the session^18^,^48^. On the first training session (CRF; continuous reinforcement sessions) one lever was extended. The lever would remain extended throughout the session, and a single lever press would deliver a food pellet (maximum of 50 pellets earned within 30 min). In some cases, food pellets were placed behind the lever to promote lever pressing. After successful completion of the CRF training rats were trained to lever press on the opposite lever using the same training procedure. In the 4 following days the side of the active lever was alternated between sessions. Then, rats were trained to lever press one time for a single food pellet in a fixed ratio (FR) schedule consisting in 50 trials in which both levers are presented, but the active lever is signalled by the illumination of the cue light above it. FR sessions began with extension of both levers (active and inactive) and illumination of the house light and the cue light over the active lever. Completion of the correct number of lever press led to a pellet delivery, retraction of the levers and the cue light turning off for a 20 s ITI. Rats were trained first with one lever active and then with the opposite lever active in separate sessions (in the same day). In a similar manner, rats were then trained using an FR4 reinforcement schedule for 4 days and a FR8 for 1 day. On the test day, rats were exposed to PR or FR experimental sessions (one session per day) according to the following schedule: day 1—FR4 (left lever); day 2—PR (left lever); day 3—FR4 (left lever); day 4—FR4 (right lever); day 5—PR (right lever); which was previously shown to elicit stable behaviour^18^,^48^. FR4 sessions were identical to FR4 sessions described above. Food rewards were earned on an FR4 reinforcement schedule during FR sessions that consisted of 50 trials. PR sessions were identical to FR4 sessions except that the operant requirement on each trial (*T*) was the integer (rounded down) of 1.4^(*T*–1)^ lever presses, starting at 1 lever press. PR sessions ended after 15 min elapsed without completion of the response requirement in a trial.

For the sessions with optical stimulation/inhibition, before the PR session began, rats were connected to an opaque optical fibre in the NAc through previously implanted cannula guide. Optical fibres were removed after each session. At the beginning of each trial of the PR session—when the retractable levers are exposed to the animal together with the cue light—animals received an optical stimulation.

Optical stimulation was performed as follows: 473 nm; frequency of 40 Hz; 12.5 ms pulses over 1 s (50% duty cycle); 10 mW at the tip of the implanted fibre. Other alternative stimulation protocols were also used: (1) 473 nm, constant light delivery over 1 s, 10 mW at the tip of the implanted fibre; (2) frequency of 10 Hz, 50 ms pulses over 1 s (50% duty cycle), 10 mW at the tip of the implanted fibre; (3) frequency of 20 Hz, 20ms pulses over 1 s (50% duty cycle), 10 mW at the tip of the implanted fibre.

Optical inhibition was performed as follows: 589 nm; 10 s constant light; 15 mW at the tip of the implanted fibre. Other additional inhibition protocols were also used: (1) 589 nm; 10 s constant light, 5 mW at the tip of the implanted fibre) 589 nm, frequency of 40 Hz, 12.5 ms pulses over 10 s.

*Rat PIT test*. Pavlovian training comprised nine daily sessions in which each of two auditory conditioned stimulus (CS, tone and white noise) was paired with a different outcome (pellet or sucrose solution). Each of the CS exposure that lasted for 2 min was presented four times per session using a pseudo randomized order, with an ITI of 2 min in average. Data were plotted as the number of magazine visits performed during both CS presentations (16 min in total) and the number of magazine visits performed during the ITI (pre-CS period). Animals were then trained for the instrumental conditioning. Training was performed in two separate sessions per day (one session for each lever) and the order of training was alternated during days (average interval between the two sessions was 3 h). Each session finished after 30 rewards were delivered or 30 min had elapsed. In the first 2 days, lever pressing was in a CRF order. The probability of getting a reward decreased according to the following sequence: days 3**–**4, random ratio (RR) 5; days 5–6, RR10. The number of lever presses per minute per session was registered and plotted. Twenty-four hours later, subjects were placed in the operant chamber to test for PIT transfer with both levers inserted. After a baseline performance interval (BPI) that lasted for 8 min, four blocks of each auditory CS were presented randomly and lever presses were registered. During each stimulus presentation, lever presses were considered correct if it encoded the same reward as the audible sound. When encoding was different, the actions were considered incorrect. The number of lever presses performed during the test is plotted. Same—lever pressing on the lever that originates the same reward as the CS presented; dif—lever pressing on the lever that originates a different reward as the CS presented.

For the sessions with optical stimulation, before the PIT session began, rats were connected to an opaque optical fibre for optical stimulation in the NAc through previously implanted cannula guide. Optical fibres were removed after each session. At the beginning of each CS presentation, rats received an optical stimulation (473 nm; frequency of 40 Hz; 12.5 ms light pulses over 1 s (50% duty cycle); 10 mW at the tip of the implanted fibre).

*Rat locomotor activity*. Locomotor behaviour was investigated using the open field test. Briefly, rats were attached to an optical fibre connected to a laser (473 or 589 nm) and immediately placed in the centre of an arena (Med Associates) and their locomotion was monitored online over a period of 40 min. Optogenetic stimulation was given every 150 s with the following conditions: 40 Hz, 12.5 ms light pulses over 1 s; 10 mW at the tip of the implanted fibre. Optogenetic inhibition was given every 150 s with the following conditions: 10 s constant light delivery; 15 mW at the tip of the implanted fibre. Total distance travelled was used as indicator of locomotor activity.

The duration and the number of stimulations given (15 stimulations) of locomotor activity protocol were matched to the number of stimulations given during the PR test session.

*Rat food consumption test*. Food intake tests were conducted in a familiar chamber containing bedding on the floor in which rats had serial access to pre-weighed quantities of regular chow pellets (20–22 g; 4RF21, Mucedola SRL) and palatable food pellets (20–22 g; F0021, BioServ) while also having constant access to water. Each food intake session consisted of 20 min access to 20 g of regular chow followed by 20 min of access to 20–22 g of palatable food pellets and chow. Laser stimulation was given once each 60 s period (stimulation: 40 Hz, 12.5 ms pulses over 1 s, 10 mW at the tip of the implanted fibre; inhibition: 10 s constant light, 15 mW). Intake tests were repeated on 3 consecutive days. Laser stimulation was administered only on 1 day, which occurred on either day 2 or 3 (counterbalanced across rats). Control intake was measured in the absence of any laser stimulation on the 2 remaining days (day 1 and either day 2 or 3, averaged together to form a baseline measurement). Chow and palatable food pellets were reweighed at the end of the test to calculate the amount consumed.

*Mouse behaviour and apparatus*. Mice were housed in groups of three animals per cage and placed and maintained on food restriction to maintain 90–95% free-feeding weight. 20 mg food pellets (F0071; BioServ), used in the behavioural protocol, were placed in their home cages on the day before the first training session to familiarize the mice with the food pellets. Behavioural sessions were performed in operant chambers (Med Associates). Each chamber contained a central, recessed magazine that provided access to 20 mg food pellets, delivered by a pellet dispenser, two ultra-light retractable levers with cue lights located above them that were located on each side of the magazine. Magazine entries were measured automatically by an infrared beam located at the entry of the magazine. A 2.8 W, 100 mA house light positioned at the top centre of the wall opposite to the magazine provided illumination. A computer equipped with Med-PC software (Med Associates) controlled the equipment and recorded the data.

*Mouse PR*. Mice were initially trained to press the lever on a FR1 reinforcement schedule whereby a single lever press elicited the delivery of a food pellet to the receptacle. One lever was designated as ‘active' (triggering delivery of food reward) and the allocation of right and left levers was counterbalanced between mice. Each trial was separated by a 5 s time-out. Each session lasted 30 min or until animals earned 30 pellets. After 9 days of FR1 training, mice performed 3 sessions of FR5. The mice were then trained in the PR schedule of reinforcement. The response ratio schedule during PR testing was calculated using the following formula (rounded to the nearest integer): =[5*e*^(R*0.2)^]-5, where *R* is equal to the number of food rewards already earned plus 1 (that is, next reinforcer). PR sessions lasted a maximum of 1 h. Failure to press the lever in any 10 min period resulted in termination of the session, which was not observed in any animal.

Light stimulation was given on one single session with the following stimulation protocol: 473 nm; frequency of 40 Hz, 12.5 ms pulses over 1 s (50% duty cycle), 10 mW at the tip of the implanted fibre. Other alternative stimulation protocol was also used: 473 nm; frequency of 20 Hz, 25 ms pulses over 1 s (50% duty cycle),10 mW at the tip of the implanted fibre.

*Mouse locomotor activity*. Locomotor behaviour was investigated using the open field test. Briefly, mice were attached to an optical fibre connected to a laser (473 nm) and immediately placed in the centre of an arena (Med Associates) and their locomotion was monitored online over a period of 40 min. Optogenetic stimulation was given every 150 s (15 stimulations) with the following conditions: 40 Hz, 12.5 ms light pulses over 1 s (50% duty cycle); 10 mW at the tip of the implanted fibre. Total distance travelled was used as indicator of locomotor activity.

*Immunohistochemistry*. For c-fos activation analysis, all animals were submitted to either the PIT or PR protocols described above. On the test day half of the animals of each group performed the test, whereas the other half did not perform the test. Rats that performed the test were sacrificed 90 min after initiation of testing and the rats that did not perform the test were sacrificed on the same day. Both groups were anaesthetised with pentobarbital (Eutasil) and transcardially perfused with 0.9% saline followed by 4% paraformaldehyde. Brains were processed and sectioned coronally on a vibratome at a thickness of 50 μm. Briefly, free-floating sections were pretreated with 3% hydrogen peroxide, rinsed in phosphate-buffered saline, blocked with 5% fetal bovine serum for 2 h at room temperature and incubated overnight at room temperature with rabbit anti-c-fos (1:1,000, Ab-5, Merck Millipore) polyclonal antibody. Afterwards, sections were washed and incubated with the appropriate secondary biotinylated antibody for 2 h, processed with an avidin–biotin complex solution and detected with 0.5 mg ml^−1^ 3,3′-diaminobenzidine. Sections were washed and mounted on glass slides, air-dried, counterstained with hematoxylin and coverslipped with Entellan (Merck). Estimation of cell density was obtained by crossing cell number values with the corresponding areas, determined using an Olympus BX51 optical microscope and the StereoInvestigator software (Microbrightfield).

*Immunofluorescence*. 90 min after initiation of the PR test, rats were deeply anaesthetised with pentobarbital (Eutasil) and were transcardially perfused with 0.9% saline followed by 4% paraformaldehyde. Brains were removed and post-fixed in 4% paraformaldehyde. Coronal vibratome sections (50 μm) were incubated with the primary antibodies mouse anti-D2 receptor (1:500, B-10, Santa Cruz Biotechnology); rabbit anti-c-fos (1:1,000, Ab-5, Merck Millipore) and goat anti-GFP (1:500, ab6673, Abcam), mouse anti-D1 receptor (1:100, NB110-60017, Novus), goat anti-ChAT (1:750, AB144P, Millipore) and mouse anti-GFP (1:200, ab1218, Abcam). Appropriate secondary fluorescent antibodies were used (1:500, Invitrogen). Finally, all sections were stained with 4′,6-diamidino-2-phenylindole (DAPI; 1 mg ml^-1^). For each animal, positive cells within the brain regions of interest were analysed and cell counts were performed by confocal microscopy (Olympus FluoViewTMFV1000). As a notice, c-fos is mainly nuclear; c-fos^+^/D1^+^ (D2^+^ or ChAT^+^) cells were considered when D1, D2 or ChAT staining delimited c-fos staining. Estimation of cell density was obtained by dividing cell number values with the corresponding areas, determined using an Olympus BX51 optical microscope and the StereoInvestigator software (Microbrightfield).

### Constructs and virus preparation

eYFP, hChR2(H134R)-eYFP and eNpHR3.0-eYFP were cloned under the control of D2 dopamine receptor minimal promoter region (Drd2; ENSRNOG00000008428; Gene ID: 24318), which included 1,540 bp upstream of the first (non-coding) exon (kindly provided by Dr. Karl Deisseroth)^49^. Constructs were packaged in AAV5 serotype by the UNC Gene Therapy Center Vector Core (UNC). Cre-inducible AAV5/EF1a-DIO-hChR2(H134R)-eYFP was obtained directly from the UNC Center. AAV5 vector titres were 3.7–6 × 10^12^ virus molecules per ml as determined by dot blot.

*Surgery and cannula implantation*. Rats were anaesthetised with 75 mg kg^−1^ ketamine (Imalgene, Merial) plus 0.5 mg kg^−1^ medetomidine (Dorbene, Cymedica). Virus was unilaterally injected into the NAc (coordinates from bregma, according to Paxinos and Watson^50^: +1.2 mm anteroposterior (AP), +1.2 mm mediolateral (ML), and -6.5 mm dorsoventral (DV)) (Supplementary Fig. 9). Rats were then implanted with an optic fibre (200 μm core fibre optic; Thorlabs) with 2.5 mm stainless steel ferrule (Thorlabs) using the injection coordinates (with the exception of DV: −6.4 mm) that were secured to the skull using 2.4 mm screws (Bilaney) and dental cement (C&B kit, Sun Medical). Rats were removed from the stereotaxic frame, sutured and let to recover for two weeks before initiation of the behavioural protocols.

Mice were anaesthetised with 75 mg kg^-1^ ketamine (Imalgene, Merial) plus 1 mg kg^-1^ medetomidine (Dorbene, Cymedica). Virus was unilaterally injected into the NAc (coordinates from bregma, according to Paxinos and Franklin^51^: +1.4 mm AP, +0.85 mm ML, and −4.0 mm DV) (Supplementary Fig. 9). Mice were then implanted with an optic fibre (200 μm core fibre optic; Thorlabs) with 2.5 mm stainless steel ferrule (Thorlabs) using the injection coordinates (with the exception of DV: −3.9 mm) that were secured to the skull using dental cement (C&B kit, Sun Medical). Mice were removed from the stereotaxic frame, sutured and let to recover for two weeks before initiation of the behavioural protocols.

*In vivo single-cell electrophysiology*. Four weeks post surgery, rats were anaesthetised with urethane (1.44 g kg^−1^, Sigma). The total dose was administered in three separate intraperitoneal injections, 15 min apart. Adequate anaesthesia was confirmed by the lack of withdrawal responses to hindlimb pinching. A recording electrode coupled with a fibre optic patch cable (Thorlabs) was placed in the NAc (coordinates from bregma, according to Paxinos and Watson^50^: +1.2 mm AP, +1.2 mm ML, and −6.0 to −7.0 mm DV), using a stereotaxic frame (David Kopf Instruments) with non-traumatic ear bars (Stoeling). Other recording electrodes with fibre optic attached were placed in the VP (coordinates from bregma, according to Paxinos and Watson^50^: 0 to −0.12 mm AP, +2.3 to +2.5 mm ML, and −7 to −7.6 mm DV) and in the VTA (coordinates from bregma, according to Paxinos and Watson^50^: −5.3 mm AP, +0.9 mm ML, and −7.5 to −8.3 mm DV) (Supplementary Fig. 9). Single neuron activity was recorded extracellularly with a tungsten electrode (tip impedance 5–10 Mat 1 kHz) and data sampling was performed using a CED Micro1401 interface and Spike 2 software (Cambridge Electronic Design). The DPSS 473 nm laser system or DPSS 589nm laser system (CNI), controlled by a stimulator (Master-8, AMPI) were used for intracranial light delivery. Fibre optic output was pre-calibrated to 10-15 mW from the fibre tip before implantation. Optical stimulation was performed as follows: 473 nm; frequency of 20 Hz, 30 Hz, 40 Hz or 50 Hz; 12.5 ms pulses over 1 s, 10 mW. Optical inhibition was performed as follows: 589 nm; 10 s constant light, 15 mW.

Firing rate histograms were calculated for the baseline (60 s before stimulation), stimulation period and after stimulation period (60 s after the end of stimulation). The cells were considered as responsive or not responsive to the stimulation on the basis of their firing rate change with respect to the baseline period. Neurons showing a firing rate increase or decrease by more than 20% from the mean frequency of the baseline period were considered as responsive^52^. Spike latency was determined by measuring the time between half-peak amplitude for the falling and rising edges of the unfiltered extra-cellular spike.

### Statistical analysis

Normality tests were performed for all data analysed. Statistical analysis between two groups was made using Student's *t*-test. Two-way ANOVA was used when appropriate. Bonferroni's *post hoc* multiple comparisons was used for group differences determination.

To capture the factorial structure of brain region measures, an Exploratory Factor Analysis was performed through Principal Axis Factoring forced to three dimensions with oblimin rotation. The internal reliability of the three dimensions was measured with Cronbach's alpha. The three dimensions were then computed using the regression method. Pearson correlations were calculated to obtain the strength of the relation between the test score and the three brain regions scores. Next, a multiple linear regression was performed to identify significant predictors of test score (using the three factors as predictors).

Results are presented as mean±s.e.m. All statistical analysis was performed using IBM SPSS Statistics (v.22) and results were considered significant for *P*≤0.05.

### Data availability

The data that support the findings of this study are available from the corresponding author on request.

## Additional information

**How to cite this article:** Soares-Cunha, C. *et al*. Activation of D2 dopamine receptor-expressing neurons in the nucleus accumbens increases motivation. *Nat. Commun.* 7:11829 doi: 10.1038/ncomms11829 (2016).

## Supplementary Material

Supplementary Information Supplementary Figures 1 - 9 and Supplementary Table 1

Peer Review File
