# Supplementary material for: Activation of D2 dopamine receptor-expressing neurons in the nucleus accumbens increases motivation
Source: Nat Commun. 2016 Jun 23;7:11829. doi: 10.1038/ncomms11829 (PMC4931006; doi:10.1038/ncomms11829)
Supplement: Supplementary Information — Supplementary Figures 1 - 9 and Supplementary Table 1 [file ncomms11829-s1.pdf]

Supplementary Figure 1

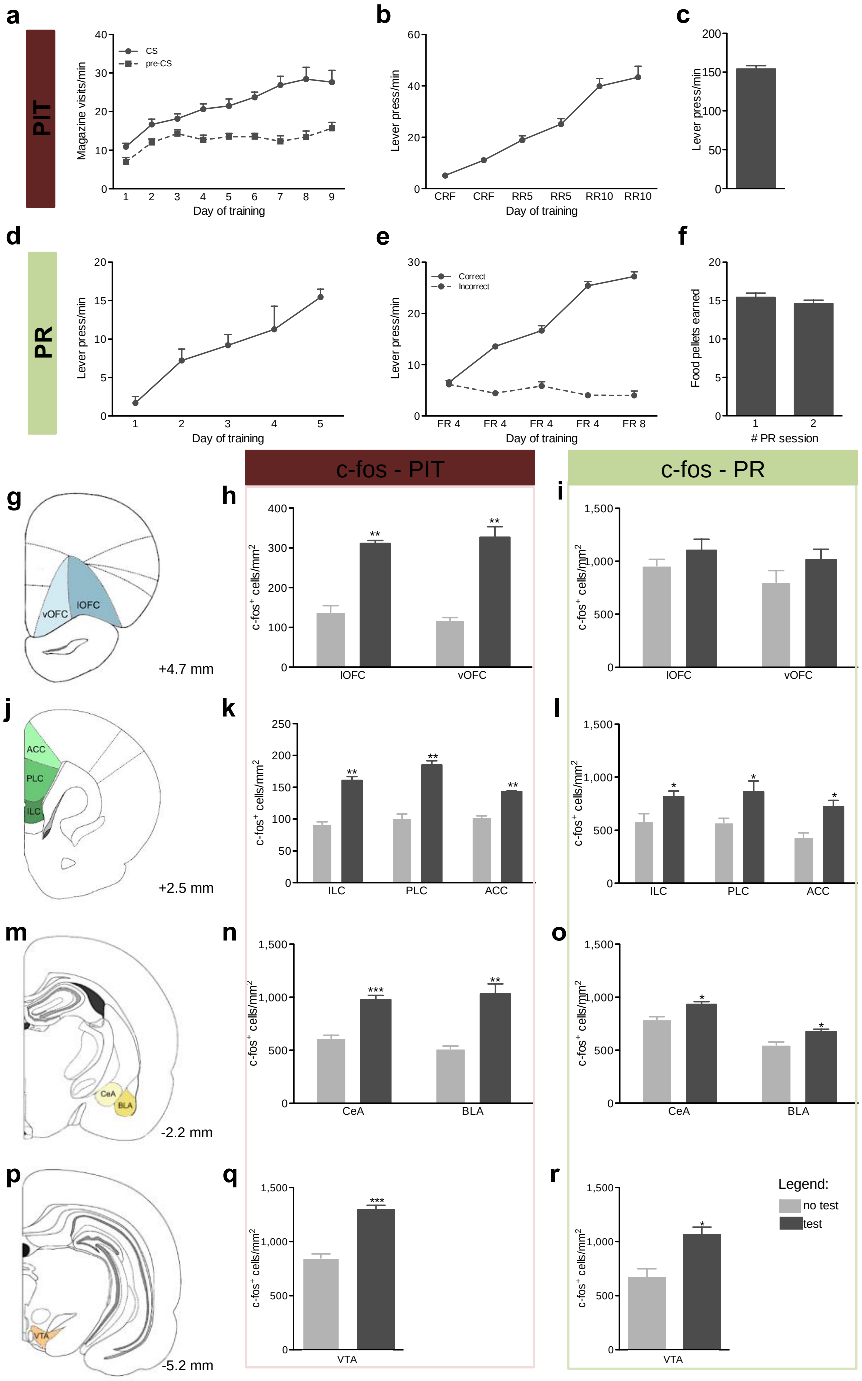

Supplementary Figure 1. Performance in two distinct motivation-dependent tasks – PIT and PR tests – and c-fos immunostaining. (**a**) Pavlovian conditioning shown as mean magazine visits per minute. (**b**) Instrumental conditioning depicted as the number of lever presses per minute performed in each training day. (**c**) Baseline performance, shown as total number of lever presses. (**d**) Continuous reinforcement (CRF) training sessions of the PR test, presented as average number of lever presses per minute. (**e**) Fixed ratio (FR) training sessions of the PR test, depicted as average number of lever presses per minute. (**f**) Average number of food pellets earned for each PR session. n=10. (**g-r**) PIT and PR induced a general activation of all cortical and limbic regions analyzed: lOFC and vOFC (**h, i**); ACC, PLC and ILC (**k, l**); BLA and CeA (**n, o**); and VTA (**q, r**) (n=6). Representative images of coronal brain sections are shown – lOFC and vOFC (**g**); ACC, PLC and ILC (**j**); BLA and CeA (**m**); and VTA (**p**); numbers represent distance in millimeters to bregma. Error bars denote s.e.m. *p≤0.05, **p≤0.01, ***p≤0.001.

Supplementary Figure 2

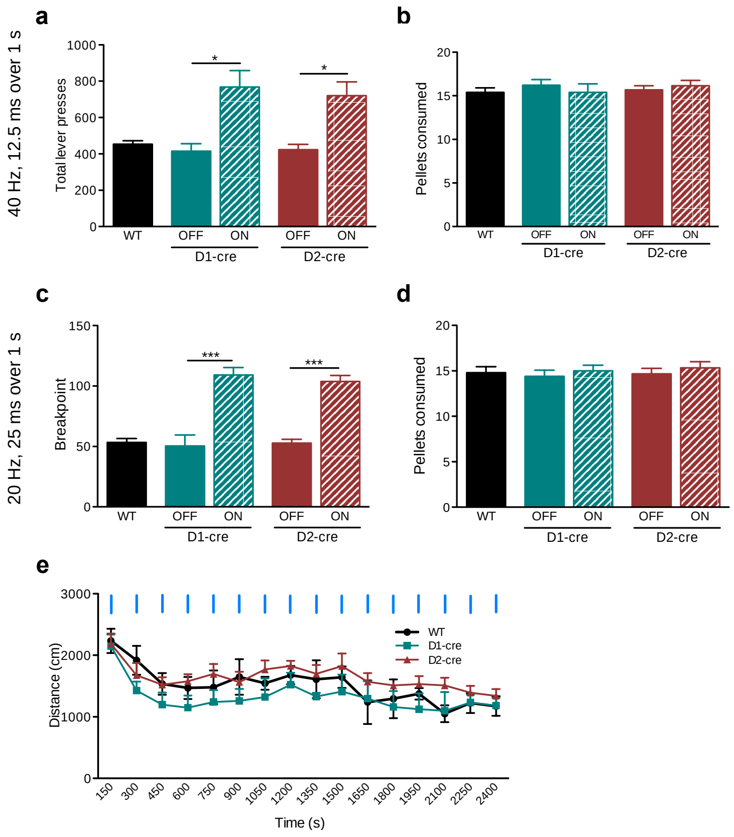

Supplementary Figure 2. Optogenetic activation of NAc D1 and D2 neurons in mice. (**a, c**) Different protocols of optogenetic stimulation of accumbal D1 and D2 neurons increased mice breakpoint ($n_{D1\text{-}cre}$=5; $n_{D2\text{-}cre}$=6). (**b, d**) No differences in the total number of pellets earned during session were found. (**e**) No major effects in locomotion of D1-cre- or D2-cre-stimulated versus non-stimulated rats. Error bars denote s.e.m. *p≤0.05, ***p≤0.001.

Supplementary Figure 3

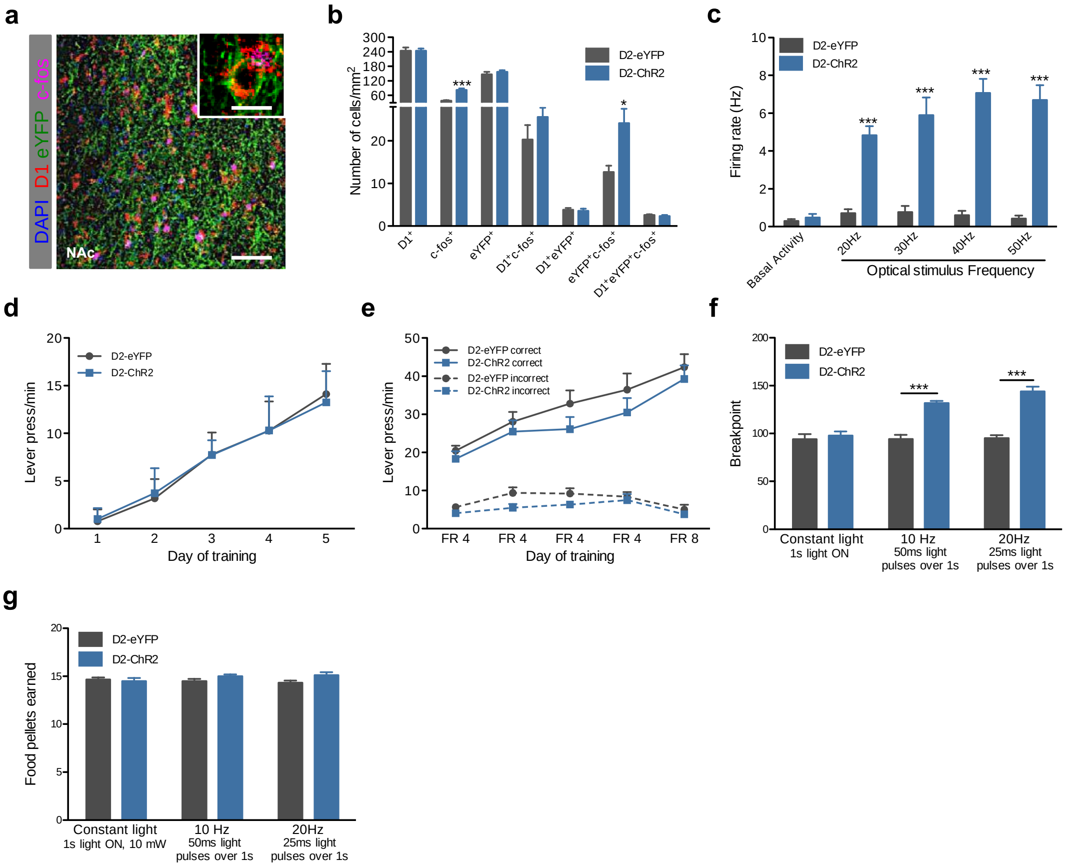

Supplementary Figure 3. Optogenetic activation of NAc D2 neurons. (**a**) Representative immunofluorescence of the NAc using antibodies against dopamine receptor D1, YFP and c-fos in an animal injected with AAV5-D2-hChR2(H134R)-eYFP that performed the PR test. Inset shows a D1$^+$/YFP$^+$ cell (bottom) and a D1$^+$/YFP$^+$/c-fos$^+$ cell (top). Scale bar: 200 µm; inset: 40 µm. (**b**) Cell counting revealed that only few D1$^+$ cells co-express YFP (average of 3.6 YFP$^+$ cells out of 244.4 D1$^+$ cells, which correspond to 1.5% of D1$^+$ neurons). No statistical difference in the activation of D1$^+$ neurons was found between stimulated D2-eYFP and D2-ChR2 animals (20.3 vs 25.62 D1$^+$/c-fos$^+$ cells; p=0.224). (**c**) D2-ChR2 opsin is functional at different stimulation frequencies (12.5 ms pulses for 1s; n_{rats}=5, $n_{neurons}$>15). (**d**) CRF training sessions of the PR schedule, shown as average number of lever presses per minute ($n_{D2\text{-}ChR2\text{-}eYFP}$=16; $n_{D2\text{-}eYFP}$=13). (**e**) FR training sessions of the PR test, presented as average number of lever presses per minute ($n_{D2\text{-}ChR2\text{-}eYFP}$=16; $n_{D2\text{-}eYFP}$=13). (**f**) Different protocols of optogenetic stimulation of accumbal D2 neurons reliably increased breakpoint with the exception of constant stimulation ($n_{D2\text{-}ChR2\text{-}eYFP}$=8; $n_{D2\text{-}eYFP}$=6). (**g**) Total number of food pellets earned in the PR session with different stimulation parameters. Error bars denote s.e.m. *p≤0.05, ***p≤0.001.

Supplementary Figure 4

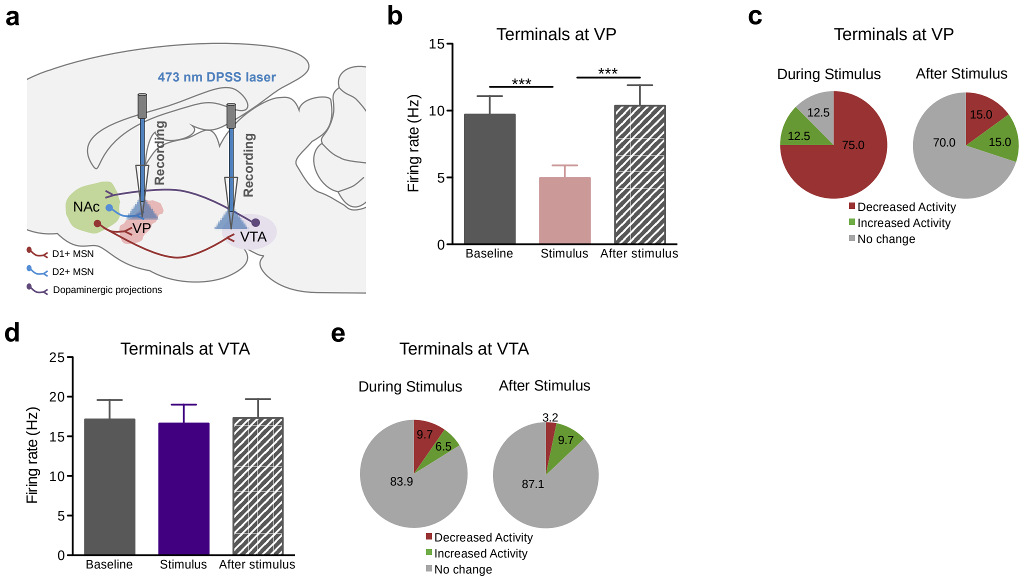

Supplementary Figure 4. Optogenetic modulation of NAc D2 terminals in the ventral pallidum (VP) and ventral tegmental area (VTA). (**a**) Schematic representation of the *in vivo* single-cell electrophysiological recording experiments with optogenetic stimulation performed in NAc terminals at VP (and VTA). (**b-c**) Stimulation of NAc terminals at the VP decreases global VP firing rate (n=30 cells), similarly to stimulation of cell bodies (depicted in Fig. 3). (**d-e**) When stimulation was performed in the VTA, no major effect in firing rate was found (n=31 cells), consistent with the absence of direct inputs to this brain region. Error bars denote s.e.m. ***p≤0.001.

Supplementary Figure 5

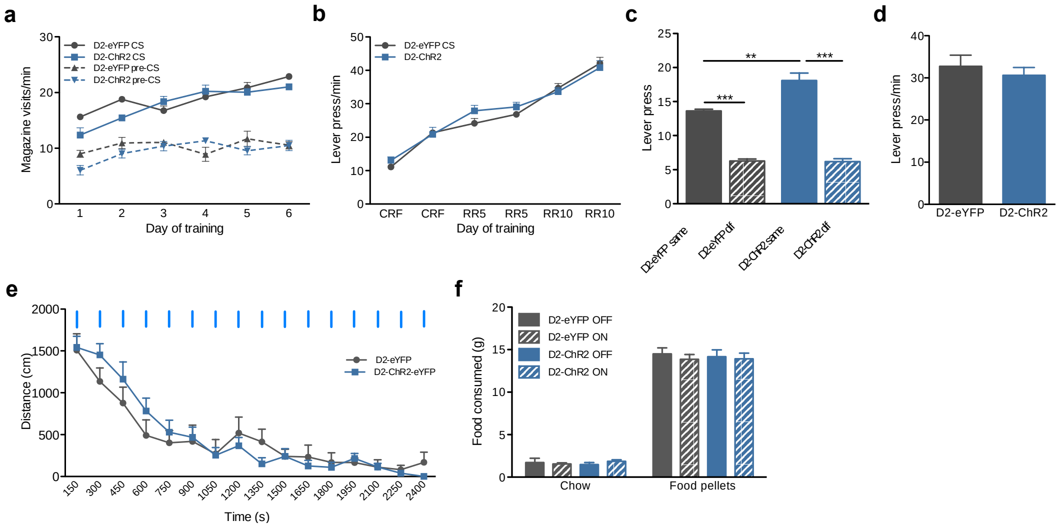

Supplementary Figure 5. Effects of NAc optogenetic activation of D2 neurons in Pavlovian-to-Instrumental Transfer (PIT) and other behaviors. (**a**) Pavlovian conditioning of the PIT test, shown as mean magazine visits per minute ($n_{D2\text{-}ChR2}$=7; $n_{D2\text{-}eYFP}$=5). (**b**) Instrumental conditioning of the PIT paradigm, depicted as number of lever presses per minute. (**c**) PIT is enhanced after NAc D2 activation (40 Hz, 12.5 ms pulses for 1 s) during conditioned stimulus (CS) presentation in D2-ChR2 animals. *Same: lever associated with CS; dif: other lever.* (**d**) Total number of lever presses in the baseline period of the PIT test. (**e**) No differences were observed in locomotor activity of D2-ChR2 stimulated versus D2-YFP-stimulated rats (40 Hz, 12.5 ms pulses during 1 s; 15 stimulations). (**f**) No effects in free feeding behavior – regular chow or sugar pellets in stimulated animals. Error bars denote s.e.m. **p≤0.01, ***p≤0.001.

Supplementary Figure 6

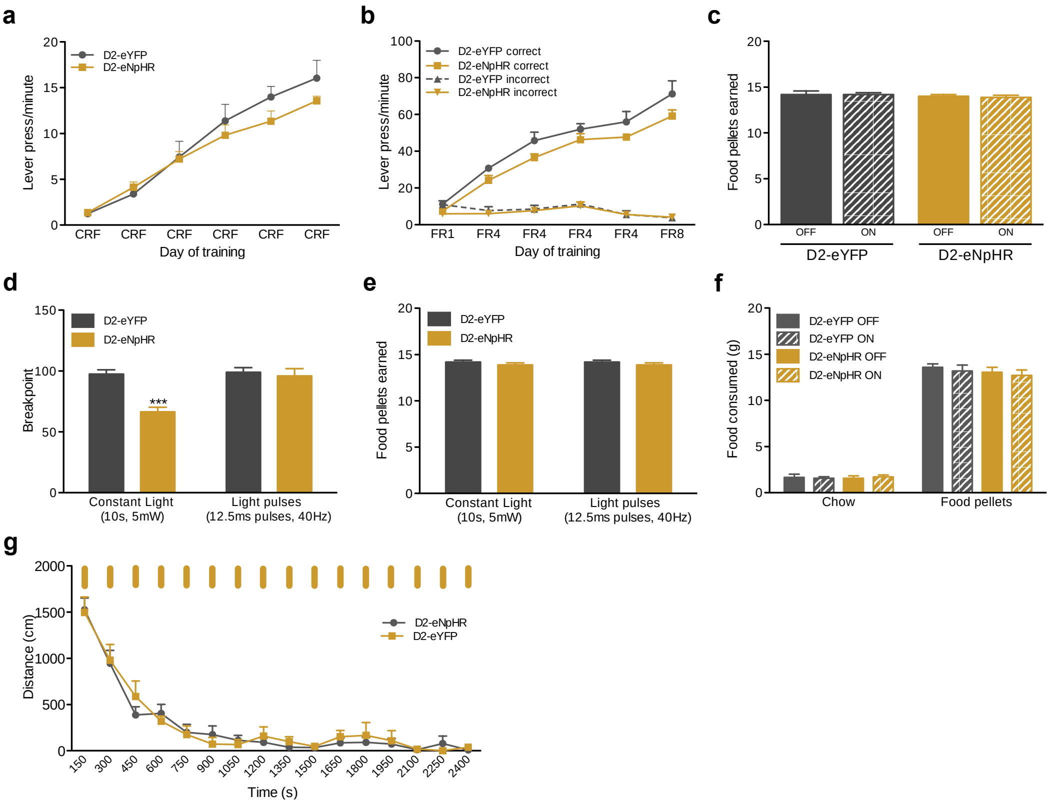

Supplementary Figure 6. Inhibition of accumbal D2 neurons - electrophysiological and behavioral correlates. (**a**) Continuous reinforcement (CRF) training sessions of the PR test is presented as average number of lever presses per minute ($n_{D2\text{-}eYFP}$=5; $n_{D2\text{-}eNpRH}$=8). (**b**) Fixed ratio (FR) training sessions of the PR test are shown as average number of lever presses per minute. (**c**) Average number of food pellets earned in the PR session with the following optical stimulation: 10 s constant light at 15 mW during cue exposure. (**d**) Breakpoint of rats that received different D2-eNpHR optical stimulation parameters Constant light at 5 mW stimulation protocol also decreased breakpoint, but not pulsed stimulation. (**e**) Average number of food pellets earned in the PR session with different optical stimulation parameters. (**f**) No major effects of D2 neuronal inhibition in free food consumption or locomotion (**g**). Error bars denote s.e.m. ***p≤0.001.

Supplementary Figure 7

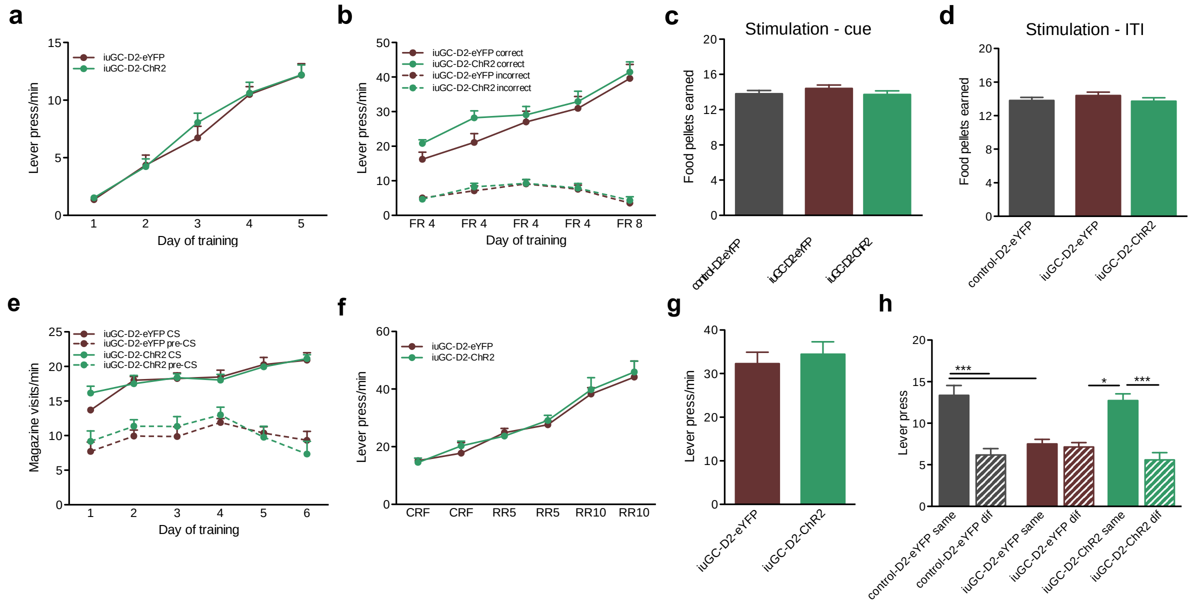

Supplementary Figure 7. Effects of NAc optogenetic activation of D2 neurons in behavior of control and iuGC animals. (**a**) Continuous reinforcement (CRF) and (**b**) fixed ratio (FR) training sessions of the PR test of iuGC-D2-ChR2 and iuGC-D2-eYFP groups. Data is presented as average number of lever presses per minute ($n_{iuGC\text{-}D2\text{-}ChR2}$=15; $n_{iuGC\text{-}D2\text{-}eYFP}$=13). (**c**) Average number of food pellets earned in the PR session of iuGC-D2-ChR2 and iuGC-D2-eYFP groups with stimulation during cue exposure. (**d**) Average number of food pellets earned in the PR session with stimulation during ITI ($n_{control\text{-}D2\text{-}eYFP}$=5; $n_{iuGC\text{-}D2\text{-}ChR2}$=7; $n_{iuGC\text{-}D2\text{-}eYFP}$=5). (**e**) Pavlovian conditioning of the PIT paradigm, shown as mean magazine visits per minute. (**f**) Instrumental conditioning of the PIT paradigm, depicted as the number of lever presses per minute. (**g**) Baseline performance of the PIT test session. (**h**) Stimulation of D2 neurons rescued PIT impairment in iuGC animals. Error bars denote s.e.m. *p≤0.05, ***p≤0.001.

Supplementary Figure 8

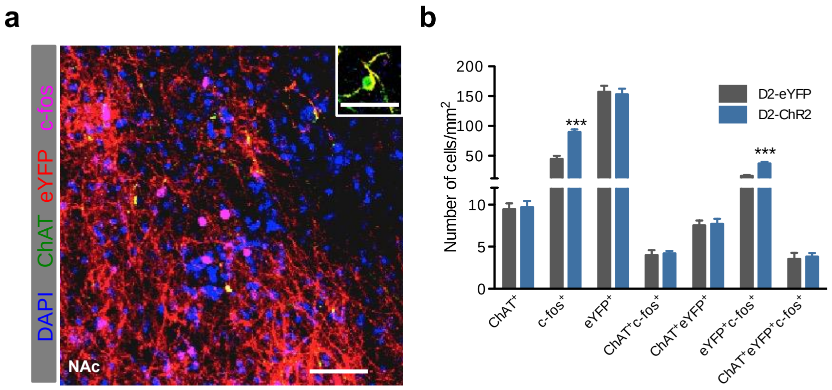

Supplementary Figure 8. Quantification of ChAT$^+$/c-fos$^+$/eYFP$^+$ neurons after D2 optogenetic stimulation during PR test. (**a**) Representative triple immunofluorescence using antibodies against YFP, ChAT and c-fos in the NAc of an animal injected with AAV5-D2-ChR2-eYFP. Inset shows a rare YFP$^+$/ChAT$^+$/c-fos$^-$ cell; scale bar: 200 μm; inset: 40 μm. (**b**) There are very few ChAT$^+$ cells per mm^2 of NAc (average of 9.5 cells/mm^2). There is no significant increase in the number of YFP$^+$/ChAT$^+$/c-fos$^+$ in stimulated D2-ChR2 animals in comparison to stimulated D2-YFP animals. Error bars denote s.e.m. ***p≤0.001.

Supplementary Figure 9

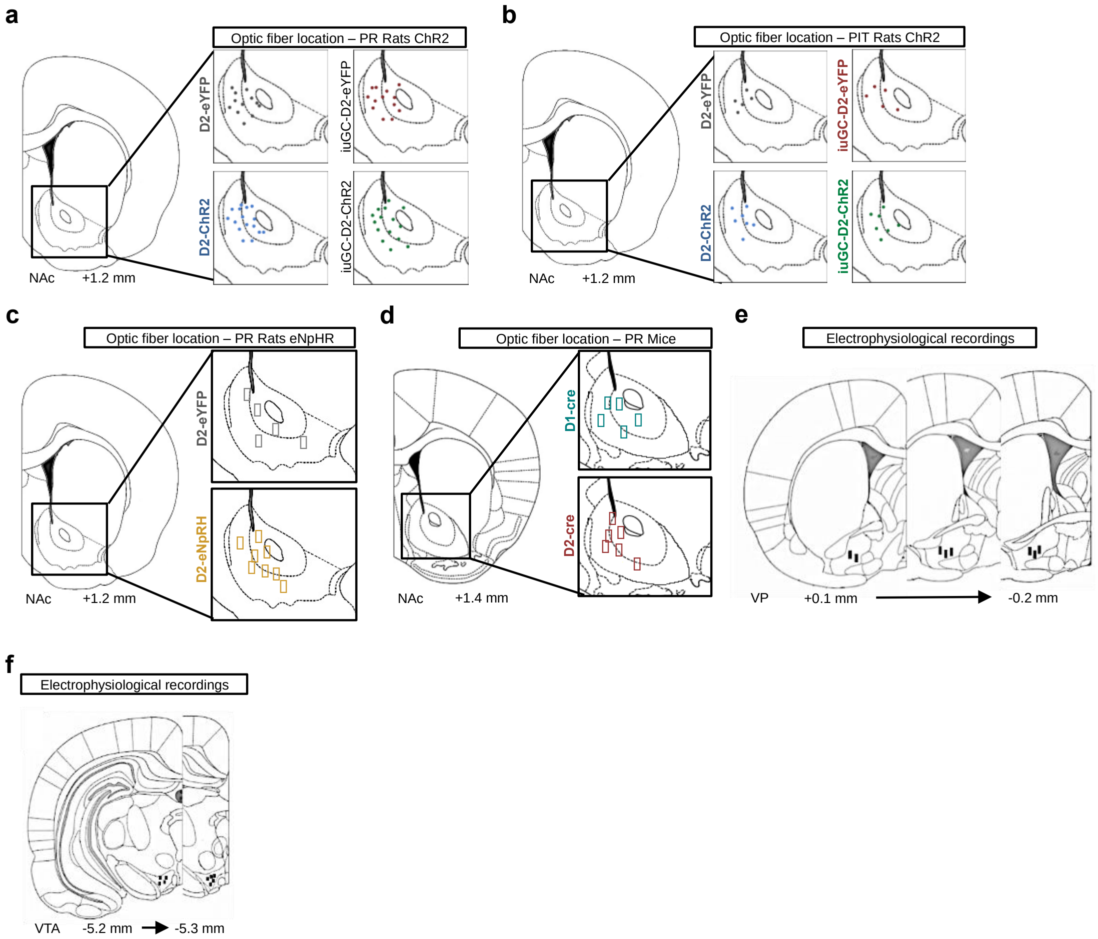

Supplementary Figure 9. Confirmation of optic fiber and electrode localization in different experiments. (**a**) Schematic representation of optic fiber placement in the NAc of rats that performed the progressive ratio (PR) paradigm with D2-ChR2 optogenetic activation. (**b**) Schematic representation of optic fiber placement in the NAc of rats that performed the Pavlovian-to-Instrumental Transfer (PIT) test with D2-ChR2 optogenetic activation. (**c**) Schematic representation of optic fiber placement in the NAc of rats that performed the PR paradigm with D2-eNpHR vector. (**d**) Schematic representation of optic fiber placement in the NAc of D1-cre and D2-cre mice + DIO-ChR2 that performed the PR test. (**e**) Schematic representation of recording electrode placement in the VP and VTA (**f**). Numbers represent distance in millimeters to bregma.

Supplementary Table 1

Supplementary Table 1. Factorial structure of brain regions analyzed by Exploratory Factor Analysis (EFA) through Principal Axis Factoring forced to three dimensions. Internal reliability assessed with Cronbach's alpha.

	Communalities		Factors (F)		
	**Initial**	**Extraction**	**F1[a.]: Cortical regions**	**F2[b.]: NAc**	**F3[c]: Other Limbic regions**
**ILC**	0.951	0.974	0.998		
**lOFC**	0.874	0.883	0.973		
**PLC**	0.958	0.946	0.933		
**vOFC**	0.822	0.799	0.849		
**ACC**	0.932	0.905	0.727		
**NAcc**	0.881	0.891		0.980	
**NAcs**	0.856	0.931		0.829	
**CeA**	0.800	0.822			0.952
**VTA**	0.802	0.839		0.315	0.694
**BLA**	0.650	0.556	-0.305		0.558
	**Cronbach's Alpha**		0.838	0.933	0.838

ACC: anterior cingulate cortex; BLA: basolateral amygdala; CeA: central amygdala; ILC: infralimbic cortex; lOFC: lateral orbitofrontal cortex; NAcc: nucleus accumbens core; NAcs: nucleus accumbens shell; PLC: prelimbic cortex; vOFc: ventral orbitofrontal cortex; VTA: ventral tegmental area.

[a.] F1, Factor 1

[b.] F2, Factor 2

[c.] F3, Factor 3
